# Organizational influences on the use of low-value care in primary health care – a qualitative interview study with physicians in Sweden

**DOI:** 10.1080/02813432.2022.2139467

**Published:** 2022-11-03

**Authors:** Gabriella Lang, Sara Ingvarsson, Henna Hasson, Per Nilsen, Hanna Augustsson

**Affiliations:** a Procome Research Group, Department of Learning, Informatics, Management and Ethics, Medical Management Centre, Karolinska Institutet, Stockholm, Sweden; b Unit for Implementation and Evaluation, Center for Epidemiology and Community Medicine (CES), Region Stockholm, Stockholm, Sweden; c Division of Society and Health, Department of Health, Medicine and Caring Sciences, Linköping University, Linköping, Sweden

**Keywords:** Low-value care, primary health care, general practitioners, organizational strategies, organizational factors, contextual factors

## Abstract

**Aim:**

The aim was (1) to explore organizational factors influencing the use of low-value care (LVC) as perceived by primary care physicians and (2) to explore which organizational strategies they believe are useful for reducing the use of LVC.

**Design:**

Qualitative study with semi-structured focus group discussions (FGDs) analyzed using qualitative content analysis.

**Setting:**

Six publicly owned primary health care centers in Stockholm.

**Subjects:**

The participants were 31 primary care physicians. The number of participants in each FGD varied between 3 and 7.

**Main outcome measures:**

Categories and subcategories reporting organizational factors perceived to influence the use of LVC and organizational strategies considered useful for reducing the use of LVC.

**Results:**

Four types of organizational factors (resources, care processes, improvement activities, and governance) influenced the use of LVC. Resources involved time to care for patients, staff knowledge, and working tools. Care processes included work routines and the ways activities and resources were prioritized in the organization. Improvement activities involved performance measurement and improvement work to reduce LVC. Governance concerned organizational goals, higher-level decision making, and policies. Physicians suggested multiple strategies targeting these factors to reduce LVC, including increased patient–physician continuity, adjusted economic incentives, continuous professional development for physicians, and gatekeeping functions which prevent unnecessary appointments and guide patients to the appropriate point of care. .

**Conclusion:**

The influence of multiple organizational factors throughout the health-care system indicates that a whole-system approach might be useful in reducing LVC.

**KEY POINTS**
- We know little about how organizational factors influence the use of low-value care (LVC) in primary health care.
- Physicians perceive organizational resources, care processes, improvement activities, and governance as influences on the use of LVC and LVC-reducing strategies.
- This study provides insights about how these factors influence LVC use.
- Strategies at multiple levels of the health-care system may be warranted to reduce LVC.

## Background

The use of low-value care (LVC) can cause patient harm and financial waste [1–5]. LVC is commonly defined as health-care activities conferring little value to patients or activities in which the risk or cost outweighs the benefits [1,6,7]. As many as 14–46% of health-care practices provided to patients are believed to be LVC [5], and 72% of physicians claim to prescribe unnecessary tests and procedures at least once a week [8]. The next 13 years will see health-care costs outpace gross domestic product growth in the Organization for Economic Co-operation and Development (OECD) countries, which makes reducing LVC critical for a more effective use of health-care resources [9].

As first-line health care, primary health care has a crucial role in the health-care system. LVC is prevalent in this setting [10–15]. In primary health care, physicians in general consider themselves cost-conscious and willing to promote the effective use of resources [16]. For example, 74% of general practitioners in a Dutch study reported that they had taken action to reduce their use of LVC [10]. However, reducing LVC in primary health care is a challenge [10–14].

Efforts have been made to identify specific LVC practices that should be reduced (e.g. Choosing Wisely) [17]. However, such lists of LVC practices are often insufficient to reduce LVC effectively [11]. Therefore, increasing attention has been paid to identifying factors that influence the use of LVC [18]. Considerable variation in the provision of LVC among individual primary care physicians indicates that differences in individual physicians’ behaviors are of great importance [19]. However, the use of LVC in primary health care can also be influenced by organizational factors [10,16,19–32], such as economic incentives [18,27–29,31], physician work culture [28], time pressure [10,16,28–30], and gatekeeping functions [28]. Thus, reducing LVC is not only individual health care professionals’ responsibility but also an issue to be considered at the organizational level [25].

In previous studies, researchers have mainly used surveys and other quantitative means of data collection to identify organizational factors that may influence the use of LVC in primary health care. In addition to this research, qualitative studies are necessary for obtaining a more in-depth understanding and exploring nuances related to *how* various organizational factors are perceived to influence the use of LVC. Improved knowledge about this matter is important to help health-care organizations and decision makers create favorable conditions for LVC reduction, which is necessary to achieve more effective use of resources. Therefore, the aim of this study was (1) to explore organizational factors influencing the use of low-value care as perceived by primary care physicians and (2) to explore which organizational strategies they believe are useful to reduce the use of LVC.

## Methods

### Design

The study was a qualitative study encompassing semi-structured focus group discussions (FGDs) about LVC with primary care physicians and was a part of a larger project [33,34]. The purpose of using this qualitative design was to obtain in-depth insights into how organizational factors may influence LVC use within primary health care.

### Study setting and context

We conducted the study in public primary health-care centers in Region Stockholm, Sweden. The staff at primary care centers in Stockholm consists of nurses, physicians and sometimes other health-care professionals such as physiotherapists, occupational therapists and psychologists working together [35–38]. Patients can choose their primary health care center and pay a smaller fee for visits [37]. Most patients are registered with a specific physician at their center. However, in a recent survey, only 60.5% of patients in Stockholm reported that they see the same physician during their visits to their health care center [39]. The Swedish health-care system consists of both private and public health-care providers. The vast majority of the privately owned health-care centers are publicly funded and most of these are owned by large, commercially oriented chains [35,36,40,41]. Publicly funded private health-care centers provide the same services and receive funding based on a combination of capitation, visits, services provided, and outcomes reached [35,38,42]. The main difference between the public and publicly funded private health-care centers is their ownership.

To achieve a large variation of settings with regard to their LVC use we recruited both high and low users of LVC. To achieve this, we held discussions with managers within health care to identify examples of LVC that could be used as a basis for the recruitment of centers. Based on these discussions, we chose low-value lab tests as our example of LVC. We used the prescription rates of four commonly used lab tests as the basis for the recruitment of centers: (1) aspartate transaminase, an enzyme found in the liver, heart, brain, pancreas, and other tissues; (2) erythrocyte sedimentation rate; (3) 25-hydroxyvitamin D3, a prohormone and the main circulating form of vitamin D; and (4) 1,25-dihydroxyvitamin D3, the biologically active form of vitamin D. The specific tests were chosen by two medical experts: a senior physician in clinical chemistry with research experience in LVC and a primary care physician with experience of quality improvement work in laboratory testing. They recommended these lab tests based on previous research [43,44] and on indications of the tests being overused within Region Stockholm.

### Recruitment of participants

We planned for six FGDs because we hypothesized that six would be sufficient to reach saturation. We based this hypothesis on a previous study by Guest et al. [45], who found that 90% of all themes were discoverable within three to six focus groups. We emailed invitations to participate to managers in all 66 publicly owned primary health-care centers in the region. We chose to restrict the study to public health care centers because of availability of data on the selected lab tests and because they provide the largest part of primary health care within the region. Representatives of 17 centers chose to participate; of those centers, we made a purposeful sample of six based on their ordering rates for the four lab tests. The subsample included centers with the lowest and highest ordering rates for at least one of the lab tests.

We contacted the six centers and scheduled FGDs, and the center managers sent invitations to the centers’ physicians. We held one FGD at each center in connection with a regular physician meeting to facilitate participation. FGDs lasted approximately 45 min on average. Thirty-one physicians participated, and the number of participants at each FGD varied between three and seven. At one of the centers, we scheduled a specific FGD time specifically for physicians who were interested in participating because the total number of physicians working at the center was too large for a focus group.

### Data collection

We chose semi-structured FGDs to capture individual physicians’ experiences as well as their shared experiences [46]. We developed an interview guide to capture thoughts about LVC as well as individual and organizational factors and strategies influencing the use of LVC practices. The data pertaining to individual factors and concerning the ways individual and system factors might interact have been published in another study [34]. The present study is based on the data from the FGDs that addressed organizational factors [34]. The questions related to practices the physicians considered LVC regardless of whether the practices were defined as such in the literature. Organizational factors were those influences that could be attributed to the organization of the health-care system, defined as the organization of people, institutions, organizations, activities, and resources to deliver health-care services [47].

The first part of each FGD focused on a broad discussion about LVC and consisted of questions exploring the participants’ general thoughts regarding LVC, including factors influencing its use and their perceptions of strategies to reduce LVC use. During the second part of the FGDs, we used the centers’ results in the four laboratory tests as examples to spur discussions offering more details about factors influencing the use of the specific LVC. During the last part of the FGDs, participants were asked what they thought could make it easier to reduce the use of LVC within health care. For more information about the interview questions, please see the supplementary material.

The second author (SI) led the interviews, and a research assistant acted as observer. The participants gave informed consent, and we informed them that participation was voluntary and that they had the right to withdraw at any time. The FGDs were audio-recorded and transcribed verbatim. Audio recording is a recommended method to document interview data within qualitative studies [48].

### Data analysis

We analyzed the data using qualitative content analysis [49]. The first and second author (GL and SI) read the transcripts several times to acquire an understanding of the content. The first author (GL) identified and condensed meaning units relevant to the aim of the study. The first author (GL) with support from the second and fifth authors (SI and HA) then summarized the condensed meaning units into codes reflecting the meaning units’ manifest meaning. The first author (GL) categorized them into subcategories, which she grouped into categories, both steps in collaboration with the other authors (SI, HH, PN, and HA). Please see Table 1 for an example of the process.

**Table 1.** Example of the analysis process.

Condensed meaning unit	Code	Subcategory	Category
Politicians rarely perform a satisfactory consequential analysis, and they often do not consider all relevant aspects when they decide what should be done.	Poor consequential analysis in decision making	Higher-level decisions	Governance

The first, second, and fifth authors (GL, SI, HA) discussed codes on several occasions and all authors took active part in discussions about categories and subcategories. The development of subcategories and categories was an iterative process with codes being regrouped throughout the analysis process to find the best possible categorization representing the data. This process continued until we reached consensus.

We selected representative quotes to reflect the physicians’ perceptions. We marked the quotations with an interviewee number (i.e. *Physician 1* being the first physician to speak during the first FDG, etc.). The physicians discussed LVC based on their own perceptions of what constitutes LVC, which may not necessarily be consistent with research-based definitions of LVC.

The research group is multiprofessional and multidisciplinary. The authors have different professional backgrounds, encompassing business and economics (GL, HH, and PN), psychology (SI), physiotherapy (HA), public health (HA) and medicine (GL). The first author (GL) is a last-year medical student without previous research experience. The second, third, fourth, and fifth authors (SI, PN, HH, and HA) have experience both with qualitative research and with research on the use and de-implementation of LVC. During the analysis we have tried to consider and scrutinize our potential preconceptions of the topic by actively questioning both ourselves and each other with regard to how our analysis of the data reflected what the physicians said during the FGDs.

## Results

Analysis of the data yielded four categories and 10 subcategories reflecting the physicians’ perceptions of organizational factors and strategies they believed could influence the use of LVC (Table 2). No new categories were identified in the last two FGDs, suggesting that data saturation was reached.

**Table 2.** The four categories and 10 subcategories.

Categories	Subcategories
Organizational resources	Time to care for patients
	Staff knowledge
	Working tools
Care processes	Work routines
	Prioritization of activities and resources
Improvement activities	Performance measurement
	Improvement work
Governance	Organizational goals
	Higher-level decisions
	Policies

### Organizational resources

#### Time to care for patients

Insufficient time during patient appointments and few available time slots for patient appointments influenced the use of LVC. Lack of time during appointments could result in physicians not performing physical examinations and an insufficient review of patients’ medical histories. These results could lead to poor-quality medical evaluations, unnecessary testing, and unnecessary referrals to other health-care providers. Another effect of lack of time was inability to communicate medical assessments and respond to patients’ questions, which in turn could increase patients’ demands for further appointments.

No one really takes the time. It’s 10 minutes at the local emergency clinic, then a visit to the emergency department and then a referral to us. Everything is quite badly organized on all levels. Instead, someone could just take the time and properly see the patient. – Physician 15, Focus Group 3

Limited available time slots for appointments influenced the use of LVC because it resulted in long waiting times. Difficulties to schedule patients for new appointments could influence physicians to prescribe medications rather than perform recommended but more time-consuming actions, such as psychological treatment or follow-up appointments. Long waiting times in primary care could also influence patients to seek emergency room care despite primary health care being the most appropriate unit for the patient’s needs.

Participants described lack of time as the result of understaffing, poor organization, and poor prioritization of health-care activities. Strategies participants suggested to overcome these problems were hiring more staff, assigning fewer administrative tasks, and implementing work methods that are more time efficient. Allowing longer patient appointments to provide patients with sufficient help at their first point of care, which could reduce the total number of visits, exemplified the latter.

#### Staff knowledge

Insufficient medical knowledge, competence, and experience among physicians could lead to poor medical decision making and therefore influence the use of LVC in patient treatment and care.

Continuous professional development as a strategy to reduce LVC was considered necessary to prevent patients from receiving outdated treatments. Participants put forth that limited teamwork in the primary health-care setting compared to many other medical specializations restricted learning and knowledge exchange with colleagues on the job. Together with insufficient time to read up on literature alone, participants believed these issues make educational activities particularly important in primary health care.

Each time I go to a course, I think that this was completely new to me. That’s a bit scary considering that there’s a huge number of things we’re expected to stay updated on. – Physician 2, Focus Group 1

Useful strategies for competence development included knowledge exchange with colleagues, training courses, and the development, improvement, and promotion of clinical guidelines. The physicians also emphasized the importance of creating a culture in which they feel safe to ask each other questions and share their mistakes for improved learning.

#### Working tools

Lack of access to effective examination tools and IT tools influenced the use of LVC. For instance, not having access to a dermatoscope (a magnifying glass used to diagnose skin conditions) caused avoidable referrals to dermatologists for symptoms that could easily have been managed within primary health care. Not having access to an emergency cabinet with emergency medicines and a defibrillator was perceived as a cause of LVC by some physicians, who considered the absence of timely and potentially life-saving basic emergency care a form of LVC.

Not having access to a patient’s previous medical record could influence the quality of medical evaluations and increase low-value testing. A suggested strategy to improve these results was additional shared IT systems with other health-care providers. Other desired strategies were additional clinical decision support to help physicians choose the best-suited imaging modality and readily available information about prices for various tests in the ordering system, which could potentially lead to fewer LVC orders.

### Care processes

#### Work routines

Insufficient continuity of care by physicians, poor patient follow-up, and poor cooperation between physicians influenced the use of LVC.

Primary health care is only about physician continuity, nothing else. By having continuity, we gain a lot of trust; all studies show that. It reduces costs, loyalty increases, morbidity and mortality decrease. – Physician 23, Focus Group 4

Continuity was considered important for primary health-care givers to follow a patient’s clinical development over time. A lack of continuity could result in suboptimal treatments as well as higher rates of ordering unnecessary tests and examinations. It could also lead to inefficient time use because patients seeking care would have to repeat their medical histories several times. As a result, participants considered physician continuity a strategy to decrease LVC. Furthermore, a trustful relationship with a patient could decrease patient demand for future unnecessary appointments and testing.

Poor follow-up and screening of patients could lead to avoidable progression of diseases and suboptimal treatments. An effective strategy used to avoid this problem was to set up ambulatory clinics for subgroups of patients, for example, patients with diabetes or hypertonia. These clinics had the benefit of clear routines and a clear division of responsibility between staff members, which contributed to both higher quality of care and more efficient follow-up.

Participants believed poor cooperation among physicians involved in a patient’s care and limited information in medical records lead to information loss as well as low-value examinations and treatments. Strategies to avoid LVC due to poor cooperation included establishing effective routines for communication and clearly dividing responsibility among cooperating physicians.

#### Prioritization of activities and resources

Participants believed problems with prioritizing health-care activities and resources influence the use of LVC. Appointments that relatively healthy patients booked, for example, for yearly health checkups or simple colds decreased access to health care and the quality of care for patients with severer symptoms. Participants mentioned unnecessary health-care activities provided to people without pressing needs as the biggest cause of LVC.

You don’t have to see the doctor for a lot of things. You can either stay at home and get healthy or perhaps visit a nurse… It sort of applies to everything, people who’ve been coughing for a few days and want blood tests because they believe they’re sick and so on. So, I think checking on healthy patients is the lowest value [task] we do; healthy people that shouldn’t be here are the ones costing the most money. – Physician 27, Focus Group 5

Participants also believed the prioritization of who or which organizations should be responsible for various health-care activities influenced LVC. They stated several activities could be performed more efficiently or better by other health-care units or professionals. Examples included non-acute visits to emergency rooms that could be managed better within primary health care and physician appointments that nurses or midwives could better manage.

Participants described several organizational factors that contribute to poor prioritization of activities and resources, for example, online booking systems that enable patients to book appointments without an assessment of their needs or the appropriate care unit. Another example was a referral rule that guarantees all patients to meet a physician within 3 days of the referral.

One strategy participants described for better prioritization of resources was to use nurses as gatekeepers who provide advice to patients, screen their needs, and guide them to the appropriate point of care. Another strategy was information campaigns about when to seek health care and which point of care to turn to in various situations.

### Improvement activities

#### Performance measurement

Data regarding, for example, the number of tests, number of prescriptions, or number of patient visits were used to evaluate the performance of both individual physicians and primary health-care centers. Depending on how the statistics were used and interpreted, these could have a positive or a negative influence on the use of LVC.

Organizational decisions based on misinterpretations of performance measures could lead to more LVC. For instance, an increased number of visits to a primary health-care center could be interpreted as a sign of increased efficiency and total health-care value produced, even if the increase was partly explained by what could be considered unnecessary visits. Physicians therefore underscored the importance of considering other potentially relevant factors, such as type of visit or patient population, when interpreting performance measures.

Participants suggested benchmarking the prescription of LVC tests as a strategy to reduce LVC. Statistics, when interpreted correctly, could correct misconceptions and create a foundation for decision making based on reality rather than assumptions, enabling improvement work to be directed toward the most pressing issues. Benchmarking could also motivate change because staff could receive feedback on their improvement and evaluate the results over time.

You shouldn’t just come and say what we ought to do but also provide feedback on what we’re actually doing. That way, it becomes a comparison. We succeed in doing that. So sometimes we do get a bit of feedback on what we’re doing. – Physician 5, Focus Group 1

#### Improvement work

The lack of improvement work at the centers was a hindrance to reducing LVC. Participants considered time restraints, absence of regular staff meetings, and short-term focus on solving acute problems the main obstacles for improvement work.

Participants gave several examples of successful internal improvement projects that had decreased LVC. One example was a project to make lab test order sets more precise for each diagnosis, thereby decreasing unnecessary testing. Another strategy was participation in externally initiated projects to reduce LVC, such as a project to improve the use of antibiotics and reduce unnecessary prescriptions to reduce antibiotic resistance. This project was described as successful due to its limited scope, continuity with information, and well-designed methods for the target groups. One internal project involving interprofessional workshops aimed at improving care processes was considered ineffective and a waste of resources because physicians saw it as having vague goals and ineffective methods. They also objected to the management making participation in it compulsory.

Participants thought a center’s size had an effect on the organization of internal improvement work. They thought a smaller staff would facilitate easy communication and flexibility and allow them to try new methods of working spontaneously. To make local work to avoid LVC more effective, physicians called for more time, structure, and continuity and better follow-up regarding implemented changes.

### Governance

#### Organizational goals

Interests among patients, politicians, and staff could lead to prioritizing organizational goals and measures that increased the use of LVC. One example was the political objectives to increase health-care access and patient satisfaction, for example, giving patients the right to receive health-care appointments within a specific time. Participants thought this measure would create unnecessary health-care appointments and examinations, decrease the accessibility of health care for the patients with the greatest medical needs, and lead to worse health outcomes overall.

The physicians thought that political decision makers and health-care managers did not sufficiently prioritize measures to decrease LVC. One strategy they suggested was that the organization would make the quality of health care a higher priority. To achieve this goal, physicians desired a stronger focus on improved physician continuity, continuing education, and a more needs-based prioritization of health-care resources.

#### Higher-level decisions

Decision making at an organization’s higher levels influenced the use of LVC when decisions resulted in inefficient coordination and prioritization of resources, poor quality of care, and ineffective LVC-reducing projects. Physicians also thought higher-level decisions contributed to the low priority of LVC-reducing strategies that they considered effective, such as continuous education. Problems with higher-level decision making included a lack of health-care knowledge among decision makers in organizational planning. Another problem was decision making based on insufficient analysis of possible negative consequences of decisions on individual health-care units and whole-system efficiency.

Influencing higher-level decisions was difficult, and little thought was given to concerns that health-care staff expressed, resulting in more LVC. Adhering to decisions that increased LVC was unavoidable, as doing so was often required for the center to receive sufficient reimbursement. Participants considered this problem deeply demoralizing.

When we’re forced to do things more or less due to badly thought-through political decisions, it’s the most demoralizing, humiliating feeling, and makes me utterly despondent – Physician 2, Focus Group 1

Participants proposed less political influence over how health-care activities were performed and prioritized as a strategy to decrease LVC. Participants believed disobedience regarding policies and guidelines perceived to increase LVC was another effective strategy. For higher-level decisions regarding organizational changes to be successful, participants emphasized the importance of gaining staff support. To achieve this goal, participants believed it was important to show staff that the proposed changes would positively affect health outcomes.

#### Policies

Central policies, for example, guidelines, payment models, procedures for referrals, and national health legislation could influence the use of LVC. Participants criticized several of these policies as ineffective in reaching their desired effects, some because they counteracted other LVC-reducing initiatives and some because they would have unintended consequences increasing the level of LVC. Some policies contributed to a growing burden of administration and unnecessary tasks, leaving less time for activities that created patient value.

Compensation rates that were higher or lower than the centers’ costs for certain activities created incentives to either overuse or underuse resources. High compensation rates for activities, for example, spirometry and physical appointments, created incentives for health-care centers to provide the activity also when the benefit was considered relatively low. This could happen at the expense of other, lower-paying activities believed to create more value. One example of this situation was the high compensation rate for physical appointments, providing an incentive to prioritize short, simple appointments for healthy patients over longer appointments for patients with greater medical needs, which require more time. As a result, centers prioritizing patients with the greatest medical needs could suffer financial deficits.

To decrease unnecessary patient visits, participants suggested making the patients take a higher part of the cost for patient visits. Another strategy to reduce the use of LVC was to adjust the payment model and remove economic incentives that encouraged certain activities over others, for example, physical appointments over phone calls. This strategy would allow physicians’ medical and economic judgement, rather than economic incentives, to play a larger role in deciding which treatment, examination method, or communication method to use. Physicians underlined the importance of economic compensation not only for providing care but also for activities that prevent unnecessary care.

…now we don’t get any compensation for that. We don’t get any money for a qualified phone call to prevent that this person books an appointment with a physician or a nurse or takes a test. Now we just have to satisfy the customer and let them come within five days. And it doesn’t matter that this appointment isn’t needed. – Physician 29, Focus Group 6

## Discussion

We found that physicians perceived four types of organizational factors—resources, care processes, improvement activities, and governance—as influencing the use of LVC in primary health care. The category of resources includes time to care for patients, staff knowledge, and working tools. Care processes refer to work routines as well as how organizations prioritize activities and resources. Improvement activities involve performance measurement and improvement work to reduce LVC. Governance includes organizational goals, higher-level decision making, and the organization’s policies. Participants suggested multiple strategies addressing these four factors to reduce LVC, including increased patient–physician continuity, adjusted economic incentives, continuous professional development for physicians, as well as gatekeeping functions whichprevent unnecessary visits and guide patients to the appropriate point of care.

### Findings in relation to prior studies

This study’s overall findings are broadly in line with previous research on organizational factors that influence LVC use. In a previous review [18], researchers identified work and care processes, policy and political support, economic incentives, and strategies as determinants for LVC. Our study adds insights into how these factors may operate to influence LVC use in primary health care.

Among the organizational resources, participants believed a perceived lack of time increased the use of LVC and hindered several LVC-reducing strategies the physicians proposed. These findings are consistent with studies indicating that time constraints commonly create LVC in primary health care [10,16,28,50–52]. Lack of time can generate LVC for multiple reasons, including physicians having limited time to inform and convince patients why a specific procedure or referral is not indicated [10,52].

The physicians suggested improved continuity of care to achieve a more efficient use of resources and a higher quality of care. Previous research provides support for the effectiveness of such a strategy, as continuity of care has been positively associated with increased time efficiency [53], fewer unnecessary medical procedures [23], lower health-care costs [54], and reduced mortality [55]. Approximately one-third of Swedish patients reported in a multi-country study that they had a regular physician or nurse [56]. This is a considerably lower proportion compared to patients reported in the study’s 10 other high-income countries, where the corresponding rate ranged from 80% to 98%. The low continuity may be explained by staff shortage, patients’ right to choose and change primary health-care centers whenever they like, and high demands on accessibility [57].

The physicians perceived it as challenging to keep updated on medical knowledge and suggested more continuing education as a strategy to reduce the use of LVC. This finding is consistent with previous research which has identified continuing education as a relevant strategy to reduce the use of LVC [6]. Nevertheless, only 47% of specialist primary care physicians in Sweden report having received education about new guidelines over the past year [58]. Furthermore, the average time spent on externally organized educational activities for physicians has decreased from 8.5 to 5.3 days in the period between 2004 and 2019. During this time, the time spent on internal and individual education has hardly changed. Contrary to several other countries [59], Sweden has no national rules regarding a minimal level of continuous education required for specialist physicians.

In line with previous studies, participants believed economic incentives influenced the use of LVC practices [28,29,31,50]. For instance, physicians said that insufficient economic compensation for activities that could reduce LVC led to them not being prioritized. In contrast, high economic compensation for certain activities provided an incentive to increase the activity’s frequency even when it was considered LVC. Furthermore, physicians provided examples of how payment models incentivized less efficient work methods and cost shifting to other parts of the health-care system. Participants believed insufficient consequential analysis regarding the effects of organizational decisions on the health-care system as a whole contributed to inappropriate incentives and organizational decisions that resulted in unintended LVC. In previous studies, researchers have emphasized the importance of applying a whole-system approach and incentive structures that adapt for positive and negative effects throughout the system to decrease the risk of unintended consequences [60–63]. Linking economic measures to measurements of LVC use and de-implementation efforts has previously been proposed as a strategy to reduce LVC [11,60]. The physicians in our study supported such a strategy and called for economic compensation not only for the care they provide but also for activities aimed at decreasing unnecessary care.

In this study, we applied a pragmatic definition of LVC and encouraged physicians to discuss practices that they considered LVC. Their understanding of LVC was broader than the research literature’s [1,6,7,^64^,^65^] definitions of the concept and encompassed more practices compared to those identified as LVC in clinical guidelines. In addition to accounting for the potential risks and expected value of various practices for individual patients, the physicians included a wide range of practices they believed decreased the total value produced by the health-care system.

These practices included time-consuming administrative tasks, unnecessary visits and health-care visits for minor medical needs because they can reduce the provision of health care for patients with greater needs. The discrepancy between what physicians perceived as LVC and what is identified as LVC practices in research may influence the extent to which physicians consider LVC a relevant problem. For example, reducing the number of unnecessary lab tests, which is defined as LVC, may be considered less important than reducing activities physicians regarded as LVC, such as the number of appointments provided to patients with minor but still existing medical needs.

In our study, physicians believed that a lack of consideration for staff concerns and a lack of health-care knowledge in higher-level management could lead to increased LVC. In a complex system such as health care, it is difficult to have an overview of and be knowledgeable about all parts of the system [66,67]. Therefore, drawing on the local staff’s knowledge is an important part of understanding how parts of the system work together [66]. Physicians and managers tend to have different worldviews, training, and loyalties [68], which can contribute to partially different understandings of problems and their possible solutions [66,68]. These differences could yield resistance to implementing changes [66]. Knowledge sharing and dialogue can help create a shared understanding of problems and their solutions, improve the likelihood of carefully considered and effective changes, and increase the engagement of those whom the changes affect [5,66].

### Implications

The primary care physicians perceived several organizational barriers to reducing LVC. However, the majority of the strategies to reduce LVC that researchers have evaluated have targeted individual health-care professionals [6,69]. Our results indicate that organizational support and relevant incentives for physicians and primary health care centers may play an important role in the implementation of LVC-reducing strategies.

Economic incentives as well as local and central policies can lead to unintentional LVC and LVC reductions across the health-care system; given these consequences, a whole-system approach is necessary. Thus, potential consequences should not be considered for only one unit but for the entire system [60–62]. Furthermore, primary care physicians could work with managers to identify the LVC activities they have, prioritize the ones that can be reduced, and develop the strategies to allow them to be reduced [5,66]. Physician-management cooperation could help enable the development and testing of physician-proposed LVC-reducing strategies, as many of their suggested strategies require higher-level support for their implementation.

### Strengths and weaknesses

A limitation is that the qualitative approach does not allow assessing associations between the organizational factors and LVC use or for determining the effectiveness of the suggested strategies to reduce the use of LVC. However, the qualitative approach provided deeper insights into *how* organizational factors influence LVC use, which is vital knowledge when developing strategies to reduce LVC.

The purposeful sampling to recruit the centers based on their prescription rates of four lab tests might narrow the results’ generalizability. However, in the first and last part of the interviews, we focused on LVC in general and allowed the physicians to discuss many examples of LVC practices other than these lab tests. Furthermore, the organizational factors described as influencing lab test use were similar to the factors related to other examples of LVC, which indicates that the results are relevant for other LVC practices in primary health care. The study setting’s characteristics should be considered when assessing the results’ transferability. The study encompassed only publicly funded centers in Stockholm. Primary health care centers in Stockholm are tax-funded based on a capitation, visits, services provided, and reached outcomes, and patients can choose which center to attend. This structure could encourage physicians to adhere to patients’ requests for various LVCs to keep patients satisfied and less likely to change providers. This physician behavior is more likely in metropolitan areas where patients have more centers to choose from compared to in rural areas. Further investigation of organizational factors influencing LVC in private centers is warranted.

## Conclusion

Our findings provide insights into how organizational factors may influence the use of LVC within primary health care. From primary care physicians’ perspectives, organizational resources, care processes, improvement activities, and governance influenced LVC use. These factors affected incentives and opportunities for the physicians and primary health-care centers to reduce LVC and implement LVC-reducing strategies. Suggested strategies to reduce LVC included increased patient–physician continuity, adjusted economic incentives, continuous professional development for physicians, as well as gatekeeping functions which prevent unnecessary visits and guide patients to the appropriate point of care. Because multiple factors throughout the health-care system influenced the use of LVC and LVC-reducing strategies, a whole-system approach might be useful in reducing LVC.

## Supplementary Material

Supplemental Material
