# Supplementary material for: Organizational influences on the use of low-value care in primary health care – a qualitative interview study with physicians in Sweden
Source: Scand J Prim Health Care. 2022 Nov 3;40(4):426–37. doi: 10.1080/02813432.2022.2139467 (PMC9848255; doi:10.1080/02813432.2022.2139467)
Supplement: Supplemental Material [file IPRI_A_2139467_SM2548.docx]

Interview guide: Focus group discussions about LVC with primary care physicians.

**The first qustion is a more general question concerning de-implementation of LVC within health care. What are your thoughts on the topic (main question in bold letters, possible follow up questions in normal letters) ?**

Positive/Negative

Barriers/ facilitators

**Is this something that you think about during your everyday work?**

What can initiate these thoughts?

If, then how do you handle it? What do you do in practice?

Have you tried to reduce the use of LVC? How did it work? What barriers/facilitators have you noticed?

Can you give any specific examples? How did it work? What did you do?

**Is this something that you work with in some way at the center?**

Could you give an example?

**Does any of you have any experience of planned interventions aiming at reducing or ceasing the use of LVC within health care? In case you have, what have they looked like? Have they succeeded? What have you done in these situations?**

Where have the decisions been made?
What kind of intervention? Education, information, instructions, feedback, problem-solving?

How have these interventions been evaluated?

Now, I would like to discuss the three lab tests that we have chosen as examples of LVC in order to learn more about LVC within your organization. The chosen lab tests are AST, vitamine D and ESR. We will shortly look at your own data concerning these tests but first I would like to hear your thoughts on defining these tests as LVC.

**What are your perspective on them being defined as LVC?**

Reasonable to try to reduce the use?

What guidelines do you have at the center concerning these tests?

**What could contribute to the use of these tests?**How do you use these tests?
What do you think may influence the use? What could influence you to use them differently?

Show the results (high, low or medium prescriber of each test):

**These are your results – What are your thoughts on the data?**

Something you recognize? Are you suprised? Reasons for the data?

**When to national board of health and wellfare publish their guidelines they also include listed practices that are called not to do – What are your thougts on these guidelines.**

**Are you aware of these guidelines?**

Positive/negative

Thoughts on how the national board of health and wellfare ought to do.

**What do you think could make it easier for personnel to reduce the use of LVC within health care?**

Organizational factors, process factors, individual factors?

Something that you would like to add that I haven’t asked about?
